# Response to ‘Increase of nerve growth factor levels in the human herniated intervertebral disc: can annular rupture trigger discogenic back pain?’

**DOI:** 10.1186/s13075-015-0607-4

**Published:** 2015-04-21

**Authors:** Mehmet Agilli, Safak Ekinci

**Affiliations:** Department of Biochemistry, Agri Military Hospital, Abide Mah, Erzuurum Cad, Agri Asker Hastanesi, Merkez, Agri Turkey; Department of Orthopaedics, Agri Military Hospital, Abide Mah, Erzuurum Cad, Agri Asker Hastanesi, Merkez, Agri Turkey

We have read with a great interest the published article by Aoki and colleagues entitled ‘Increase of nerve growth factor levels in the human herniated intervertebral disc: can annular rupture trigger discogenic back pain?’ [1]. The authors suggest that nerve growth factor (NGF) increases in herniated discs, and may play an important role in the generation of discogenic pain. However, we think some points should be discussed.

NGF is a polypeptide that plays an important role for cells belonging to the nervous, endocrine, and immune systems [2]. We think the study group in which the authors evaluated tissue NGF levels is not well defined. Namely, they should state whether the patients have one of following diseases, which could probably affect NGF levels: neuropsychiatric diseases such as epilepsy, depression, schizophrenia, migraine and primary headache; and eating disorders or cardiometabolic diseases such as atherosclerosis, metabolic syndrome and type 2 diabetes mellitus [3]. In addition to these diseases, obesity is also shown to affect NGF levels [4]. In this regard, the above diseases should be taken into account while designing a study group, and the body mass index of participants has to be thought of as another confounding variable and included in multiple regression analysis like age and sex are.

Besides the above confounders, supplements such as vitamin D analogs, zinc, vitamin B12, vitamin A, omega 3 fatty acid or herbal medicines and some drugs such as estrogen, glucocorticoids, acetyl-l-carnitine and antipsychotics also have to be denoted regarding whether the participants use these medications or not because they are affecting factors for NGF [5].

In conclusion, explanation of these concerns will certainly provide clearer information for the readers.

## Abbreviations

NGF: Nerve growth factor.
